# Evolutionary Conservation of PP2A Antagonism and G2/M Cell Cycle Arrest in Maedi-Visna Virus Vif

**DOI:** 10.3390/v14081701

**Published:** 2022-08-01

**Authors:** Adeline M. Luperchio, Stefán R. Jónsson, Daniel J. Salamango

**Affiliations:** 1 Department of Microbiology and Immunology, Stony Brook University, Stony Brook, New York, NY 11794, USA; adeline.luperchio@stonybrook.edu; 2 Institute for Experimental Pathology, University of Iceland, Keldur, 112 Reykjavik, Iceland; stefanjo@hi.is

**Keywords:** HIV-1, host-pathogen, MVV, phosphatase regulation, PPP2R5, Vif

## Abstract

The canonical function of lentiviral Vif proteins is to counteract the mutagenic potential of APOBEC3 antiviral restriction factors. However, recent studies have discovered that Vif proteins from diverse HIV-1 and simian immunodeficiency virus (SIV) isolates degrade cellular B56 phosphoregulators to remodel the host phosphoproteome and induce G2/M cell cycle arrest. Here, we evaluate the conservation of this activity among non-primate lentiviral Vif proteins using fluorescence-based degradation assays and demonstrate that maedi-visna virus (MVV) Vif efficiently degrades all five B56 family members. Testing an extensive panel of single amino acid substitution mutants revealed that MVV Vif recognizes B56 proteins through a conserved network of electrostatic interactions. Furthermore, experiments using genetic and pharmacologic approaches demonstrate that degradation of B56 proteins requires the cellular cofactor cyclophilin A. Lastly, MVV Vif-mediated depletion of B56 proteins induces a potent G2/M cell cycle arrest phenotype. Therefore, remodeling of the cellular phosphoproteome and induction of G2/M cell cycle arrest are ancient and conserved functions of lentiviral Vif proteins, which suggests that they are advantageous for lentiviral pathogenesis.

## 1. Introduction

Lentiviruses encode three universal retroviral proteins (Gag, Pol, and Env), one lentivirus-specific protein named Vif (except for equine infectious anemia virus), and other accessory factors that are less conserved. The canonical function of lentiviral Vif proteins is to counteract the APOBEC3 (A3) DNA cytosine deaminases [1,2,3,4]. In the absence of Vif, A3s package into nascent viral particles and generate C-to-U lesions in the viral cDNA. Vif neutralizes the A3s by nucleating the formation of a cullin-RING ubiquitin ligase complex to target A3 enzymes for proteasomal degradation [5,6]. Because of the potency of this restriction mechanism, A3s were thought to be Vif’s only substrates. However, recent quantitative proteomic studies identified new Vif targets, including multiple members of the B56 family of protein phosphatase 2A (PP2A) regulators [7,8].

PP2A enzymes function as heterotrimeric complexes comprised of a phosphatase enzyme, a scaffolding protein, and a regulatory (B) subunit from one of three different protein families (B55α-δ, B56α-ε, or PR72/130) [9,10,11]. The regulatory subunit dictates holoenzyme localization and substrate identification, and without it the complex is rendered non-functional [9,12]. Recently, several groups have established a direct cause-and-effect relationship between Vif-mediated depletion of B56 proteins and induction of G2/M cell cycle arrest, a previously ascribed Vif function [13,14,15,16,17,18]. The aim of the current study was to determine the conservation of Vif-induced B56 antagonism and G2/M cell cycle arrest activities in non-primate lentiviral Vif proteins.

## 2. Materials and Methods

### 2.1. Cell Culture and Cloning

HEK293FT and HeLa cells were maintained in RPMI (Hyclone, South Logan, UT, USA) and supplemented with 10% FBS (Gibco, Gaithersburg, MD, USA) and 0.5% pen/strep (50 units). Cells were transfected with PEI using a ratio of 3 μL per 1 μg of DNA. To generate the HEK293T eGFP-B56α-ε wild-type and mutant stable cell line, roughly 400,000 HEK293FT cells were seeded in a 6-well plate and transfected with a pQCXIH retroviral vector containing the relevant eGFP-B56 expression cassette [13], an MLV-GagPol packaging vector, and a VSV-G vector. Media was harvested 48 h post-transfection and frozen at −80 °C for 4–6 h, thawed and centrifuged at 1500× *g*, and overlaid on fresh HEK293T cells. To generate a pure cell population, cells were treated with hygromycin B (Sigma-Aldrich, St. Louis, MO, USA, 250 μg/mL) 48 h post-transduction. The same procedure was followed for generating *CPYA* knock-down and control cell populations with the following modifications. Scrambled and *CYPA* shRNA pLKO vectors [19], were co-transfected with an HIV-1 packaging vector and VSV-G in HEK293FT cells to generate virus. All wild-type and mutant constructs were generated by PCR amplification using Phusion high fidelity DNA polymerase (NEB, Ipswich, MA, USA) and overlapping PCR to introduce the indicated mutations. Non-primate lentiviral Vif sequences were ordered through Integrated DNA Technologies as gBlock fragments and cloned into the mTagBFP2-T2A expression construct. The following Vif isolates were used for these studies: HIV-1, NL4-3; FIV, AY600517.1; BIV, NP_040564.1; CAEV, AAG48630.1; and MVV, AAA17527.1. All constructs were confirmed by restriction digestion and Sanger sequencing.

### 2.2. RNA Isolation, cDNA Synthesis, and Inhibitor Treatments

Roughly 250,000 scrambled control or *CYPA* stable cells were collected and subjected to RT-PCR analysis. RNA was isolated from the indicated cell lines using Trizol/chloroform extraction. Briefly, 500 μL of Trizol reagent (Thermo Fisher Scientific, Waltham, MA, USA) was added to cell pellets, mixed thoroughly, and incubated at room temperature for 5 min. Then, 100 μL of chloroform was added, mixed thoroughly, and incubated at room temperature for 3 min. Samples were centrifuged at 12,000 RPM for 15 min at 4 degrees for phase separation. The RNA-containing upper phase was removed and combined with 250 μL isopropanol, mixed thoroughly, and incubated at room temperature for 2 h. Samples were centrifuged at 12,000 RPM for 15 min at 4 degrees to pellet RNA, washed once with 500 μL 70% ethanol, pelleted as described above, and allowed to air dry. Roughly 1 μg of RNA was used as input for cDNA synthesis reactions using a RevertAid Reverse Transcriptase kit (ThermoFisher) and the standard reaction mixtures. Briefly, RNA was combined with 1 μL of 100 μM oligo dT primer and incubated at 65 degrees for 5 min. Then, the RNA-primer mixture was combined with a reaction buffer, dNTPs, RNAse inhibitor (ThermoFisher), and reverse transcriptase and incubated at 42 degrees for 1 h before a 70-degree denaturation step for 10 min. For RT-PCR reactions, 0.5 μL of cDNA template was added to the following reaction mixture: 37.8 μL dH2O, 0.5 μL 10 mM dTNP mix, 10 μL 5× Phusion reaction buffer, 0.5 μL of 10 μM forward/reverse gene specific primers (*GAPDH*: Forward—5′-GAAATCCCATCACCATCTTCCAGG-3′, Reverse—5′-GAAATCCCATCACCATCTTCCAGG-3′; *CYPA*: Forward—5′-GCTGGACCAACACAAATG-3′, Reverse—5′-TCTTCTTGCTGGTCTTGCC-3′), and 0.2 μL Phusion DNA polymerase (ThermoFisher). 

For experiments using the cyclosporin A (CsA) inhibitor, roughly 150,000 HEK293FT eGFP-B56α cells were seeded into a 12 well culture plate and transfected 24 h later using PEI and 600 ng of the indicated Vif construct. After 24 h, the indicated concentration of CsA was added and incubated for 24 h. At 48 h post-transfection (24 h of CsA treatment), cells were detached using 0.25% Trypsin/EDTA and subjected to flow cytometry analysis.

### 2.3. Propidium Iodide Staining and Cell Cycle Arrest

Roughly 80,000 HeLa cells were seeded into a 12 well culture plate and transfected 24 h later with PEI and 1 μg of the indicated plasmid DNA. The cells were incubated overnight and then expanded into a 6 well culture plate to promote cell division. At 48 h post-transfection, the cells were harvested using 0.25% Trypsin/EDTA and pelleted at 5000× *g* for 10 min at room temperature. Almost all the supernatant was decanted from samples and the pellets were resuspended in the remaining liquid (~50 μL) prior to fixing in 300 μL of ice cold 70% ethanol. The samples were fixed for at least 30 min on ice before pelleting at 5000× *g* for 10 min at room temperature. The cell pellets were washed 2× in 300 μL ice cold PBS. After washing, the samples were pelleted at 5000× *g* for 10 min at room temperature and re-suspended in 500 μL of FXCycle PI stain (Invitrogen, Waltham, MA, USA), mixed well, and then analyzed via flow cytometry without washing out the dye. 

### 2.4. Fluorescence Microscopy

Roughly 15,000 HEK293T cells were seeded into an 8-chamber glass bottom slide (Ibidi) and allowed to adhere overnight. The next day, the cells were transiently transfected with 75 ng of the indicated Vif plasmid and 150 ng of the indicated B56α plasmid, and imaged on an EVOS M5000 fluorescence microscope 48 h post-transfection. Images were acquired using a 60× oil-immersion objective.

### 2.5. Flow Cytometry

All flow cytometry experiments were repeated 3 independent times and representative histograms are depicted from one experiment. Quantification of the fluorescence intensity was performed using a Becton Dickinson FACScan flow cytometer. Roughly 150,000 HEK293T cells stably expressing the indicated protein were seeded into a 12 well culture plate and transfected 24 h after plating with PEI and 600 ng of the indicated DNA. At 48 h post-transfection, the cells were removed from the plates using 0.025% Trypsin/EDTA solution, centrifuged at 300× *g* for 5 min, and then re-suspended in 2% FBS in PBS. The samples were subjected to flow cytometry and analyzed using FloJo software.

For testing B56α single amino acid substitution mutants, 150,000 HEK293FT cells were seeded into 12 well culture plates and transfected with 300 ng of the indicated B56α mutant and 300 ng of the indicated Vif construct 24 h after plating. At 48 h post-transfection, the cells were detached using 0.25% Trypsin/EDTA and subjected to flow cytometry analysis.

### 2.6. Statistical Analyses and MVV Vif Modeling

All bar graphs and statistical analyses were performed using GraphPad Prism software. Statistical significance was determined using an unpaired two-tailed student’s *t*-test. To generate a partial E3 ubiquitin ligase complex, Chimera software was used to position *CYPA* (PDB: 1CWA) over the MVV Vif PP21,24 motif and ELOB/C (PDB: 4N9F) over the SLQ BC-binding box. Surface representations of all three proteins were used to minimize steric clashing. The Phyre2 protein modeling server was used to generate a MVV Vif model with roughly 30% confidence based on multiple template segments that ranged from 25–85% amino acid identity. The WebLogo was created using an open access web portal. Phylogenetic analysis of MVV Vif isolates was performed using Clustal Omega software.

## 3. Results

### 3.1. B56α-ε Degradation Activity Is a Conserved Function of MVV Vif

**Figure 1:**
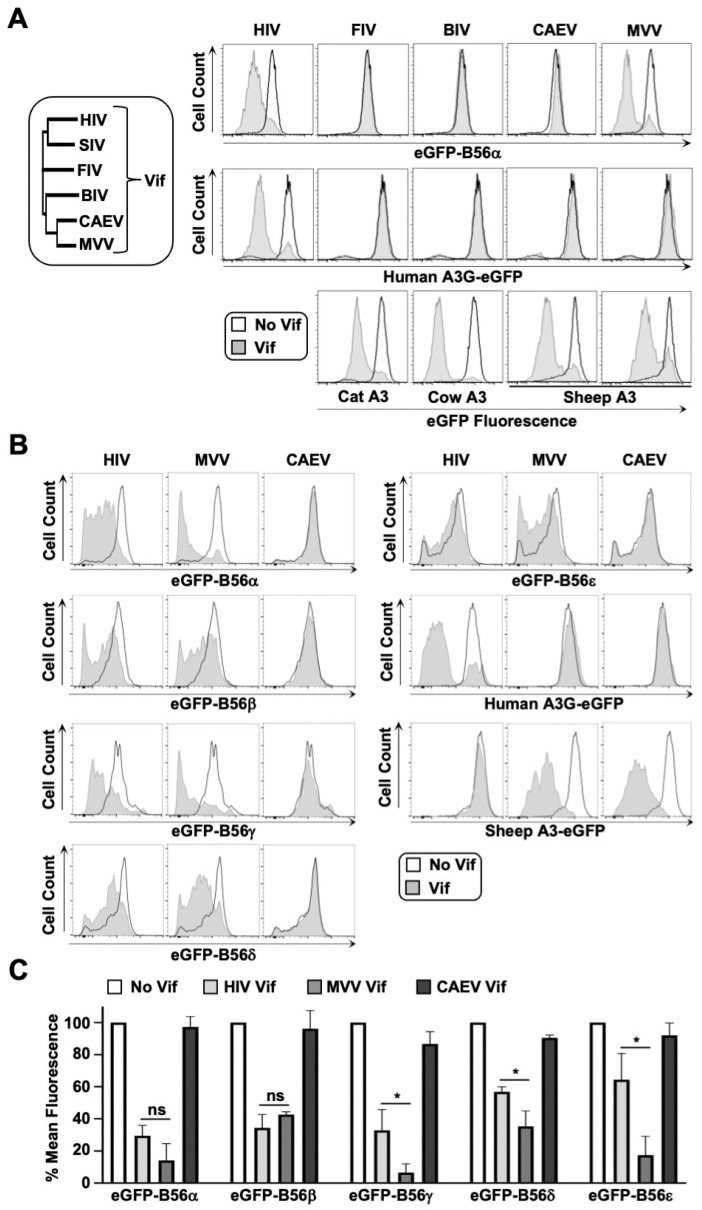
MVV Vif efficiently targets the family of B56α-ε proteins for degradation. (**A**,**B**) low cytometry histograms of A3-eGFP or eGFP-B56α degradation in the presence of the indicated Vif protein. The open histogram (black) represents the eGFP profile of cells expressing mTagBFP2 alone, whereas the filled histogram (grey) represents the profile of cells expressing the indicated Vif construct. The data shown are from one of three independent experiments. (**C**) Quantification of eGFP mean fluorescence intensity from cells expressing the indicated Vif proteins. ns, indicates no significance; * is *p* < 0.05 by unpaired student’s *t*-test. All experiments were repeated at least three independent times.

Previous studies have established that diverse HIV-1 and SIV isolates can efficiently target B56α-ε phospho-regulators for proteasomal degradation [7,13,14,20]. Here, we wanted to evaluate the conservation of this activity among non-primate lentiviral Vif proteins (phylogenetic analysis of Vif sequences depicted in Figure 1A, left). Vif isolates from feline immunodeficiency virus (FIV), bovine immunodeficiency virus (BIV), caprine arthritis encephalitis virus (CAEV), and maedi-visna virus (MVV) were cloned into a mTagBFP-T2A-Vif expression plasmid and transiently expressed in HEK293T cells stably expressing eGFP-tagged B56α, and degradation was assessed via flow cytometry (i.e., loss of eGFP fluorescence in mTagBFP positive cells). To control for non-specific degradation activity and to verify that expression constructs were functional, each Vif isolate was tested against human and cognate APOBEC3 (A3) proteins, respectively (Figure 1A, right). While all Vif isolates demonstrated robust degradation activity against their cognate eGFP-tagged A3 substrates (Figure 1A, bottom), only HIV-1 and MVV Vif proteins demonstrated activity against human eGFP-B56α, which was interesting given that HIV-1 and MVV Vif only share ~15% amino acid identity (Figure 1A, top). Only HIV-1 Vif could degrade human A3G-eGFP, which was expected given that host–pathogen interactions are typically restricted to lineage-specificity (Figure 1A, middle).

We next asked whether MVV Vif could recognize and degrade additional B56 family members. Cell lines were generated that stably expressed eGFP-tagged versions of each B56 family member (α, β, γ, δ, ε) and degradation efficiency was compared between HIV-1 and MVV Vif proteins (Figure 1B). Surprisingly, MVV Vif induced robust degradation of all five B56 proteins, and in several instances, was significantly more efficient at degrading these substrates compared to HIV-1 Vif (Figure 1C). Consistent with the observations from Figure 1A, Vif from closely related CAEV failed to degrade B56α-ε proteins, suggesting that B56 antagonism may be specific to the sheep lineage and that B56 proteins are bona fide MVV Vif substrates. Furthermore, these results support the findings of a previous study that demonstrated MVV Vif could degrade HA-tagged B56α-ε proteins [7].

### 3.2. CYPA Is Required for MVV Vif-Induced Degradation of B56 Proteins

MVV Vif’s ability to efficiently deplete all five human B56 proteins was somewhat surprising given its minimal sequence conservation to HIV-1 Vif and that it requires a distinct cellular cofactor for activity [21,22]. While all lentiviral Vif proteins nucleate the formation of cullin-RING ubiquitin ligase complexes, some also require a lineage-specific cellular cofactor for activity [6,21]. HIV-1 Vif requires the cellular protein CBFβ to promote degradation of A3 and B56 substrates [6,7,23], while MVV Vif requires the prolyl isomerase cyclophilin A (*CYPA*) to degrade cognate A3 proteins [6,21,22]. 

**Figure 2:**
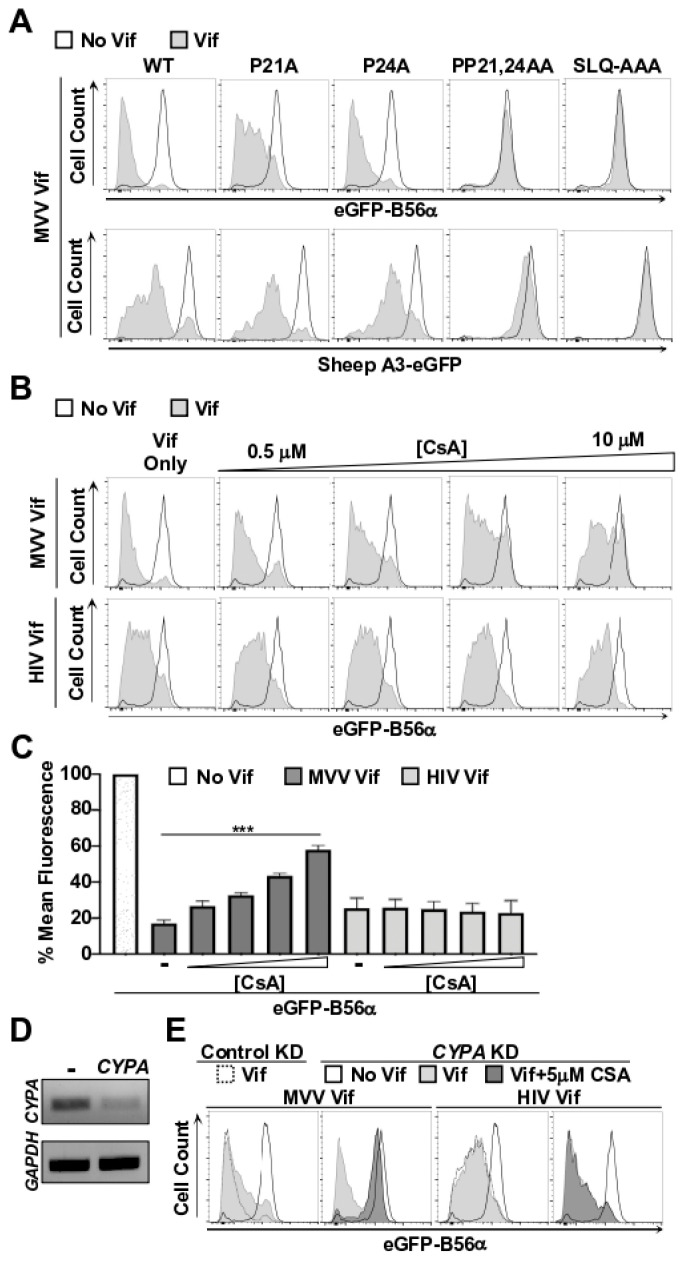
*CPYA* is required for MVV Vif-induce degradation of B56 proteins. (**A**,**B**) Flow cytometry histograms were generated as described in Figure 1. For cyclosporin A (CsA) treatments, the indicated concentration of inhibitor was added to samples transiently expressing either HIV or MVV Vif proteins. (**C**) Quantification of eGFP mean fluorescence intensity from cells expressing the indicated Vif proteins with or without increasing concentration of CsA treatment. *** is *p* < 0.001 by an unpaired student’s *t*-test. (**D**) RT-PCR analysis of HEK293FT eGFP-B56α cells stably expressing the indicated shRNA control or *CYPA* knock-down constructs. (**E**) Flow cytometry histograms were generated as described in Figure 1. Dashed lines represent eGFP-B56α cells expressing a shRNA control vector and solid lines represent eGFP-B56α cells expressing a *CYPA* knock-down construct. All experiments were repeated at least three independent times.

Here, we wanted to determine if *CPYA* is also required for MVV Vif-induced degradation of B56 substrates. It has been established that MVV Vif binds the catalytic active site of *CPYA* through proline residuesat positions 21 and 24, and that mutating these residues ablates A3 degradation activity [21]. Therefore, we generated single and double amino acid substitution mutations at these positions and assessed B56α degradation activity. Substitution of either P21A or P24A alone had minimal impact on the degradation efficiency of A3 or B56 substrates (Figure 2A). However, the PP21,24AA double mutant completely blocked degradation activity, which mirrored a SLQ-AAA mutant that is defective for recruiting the E3 ubiquitin ligase complex (Figure 2A). These observations are consistent with a previous study that determined that the mutation of both proline residues was required for full inhibition of A3 degradation activity [21], and suggest that *CPYA* is required for degradation of both A3 and B56 substrates. 

To independently verify that *CPYA* is required for B56α degradation activity, we used a combination of pharmacologic and genetic approaches to disrupt the MVV Vif-*CPYA* interaction. First, we tested the ability for the chemical inhibitor cyclosporin A (CsA), which binds the *CPYA* active site, to inhibit MVV Vif-induced degradation of B56α. We hypothesized that if *CPYA* is indeed required for B56α degradation, then CsA would compete for *CPYA* binding and decrease B56α degradation efficiency. Consistent with this reasoning, addition of CsA to cells expressing MVV Vif caused a corresponding increase in B56α-eGFP fluorescence intensity in a dose-dependent manner (Figure 2C,D). Importantly, increasing concentrations of CsA had no discernable impact on HIV-1 Vif-induced degradation of B56α-eGFP, indicating that CsA does not generally inhibit E3 ubiquitin ligase activity (Figure 2C,D). 

While these results further implicated *CPYA* as being required for MVV Vif-induced degradation of B56α, CsA treatment did not fully restore eGFP fluorescence intensity (only ~60% restoration). Therefore, we predicted that short-hairpin RNA (shRNA)-mediated knockdown of *CYPA* would result in stronger inhibition of Vif-induced degradation of B56α. To test this idea, we stably introduced previously published control and shRNA constructs targeting *CYPA* into B56α-eGFP cells and assessed MVV Vif-induced degradation [19]. Although strong *CYPA* knockdown could be achieved (Figure 2E), we only observed a modest inhibition of B56α-eGFP degradation when MVV Vif was transiently expressed in this cell line (Figure 2F). However, combining CsA treatment with *CYPA* knockdown fully ablated MVV Vif-mediated degradation of B56α−eGFP, but had no impact on HIV-1 Vif-induced degradation (Figure 2F). Taken together, these observations strongly implicate *CPYA* as being critical for MVV Vif-induced degradation of B56 substrates. 

### 3.3. HIV and MVV Vif Bind Partially Distinct B56 Surfaces through Clustered Electrostatic Interactions

**Figure 3:**
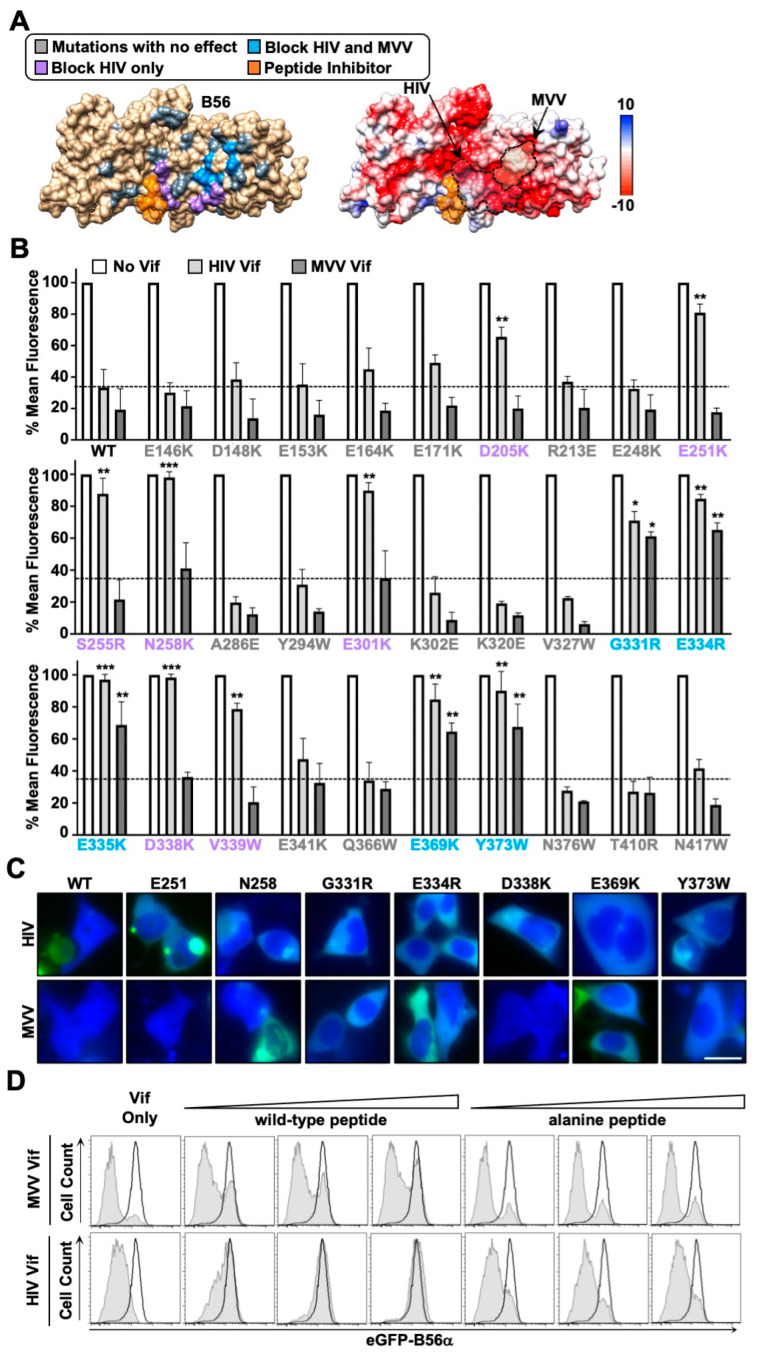
HIV and MVV Vif proteins partially distinct negatively charged B56 surfaces. (**A**) Left, surface depiction of B56γ (PDB: 2IAE) with residues required or dispensable for Vif degradation highlighted in the indicated color. Grey, no impact to degradation; purple, inhibit HIV Vif-induced degradation; blue, inhibit both HIV and MVV Vif-induced degradation; orange, peptide inhibitor binding site. Right, electrostatic surface potential map with red indicating negative charge, white indicating neutral charge, and blue indicating positive charge. Vif binding surfaces depicted by dashed lines (**B**) Quantification of eGFP mean fluorescence intensity from eGFP-B56α cells expressing the indicated Vif proteins. No statistical value indicates no significant difference between respective wild-type and mutant Vif proteins; * is *p* < 0.05; ** is *p* < 0.01; *** is *p* < 0.001 by an unpaired student’s *t*-test. (**C**) Fluorescence microscopy images of HEK293T cells transiently expressing the indicated Vif proteins and B56α proteins; scale bar is 10 μM (**D**) Flow cytometry histograms were generated as described in Figure 1. Cells were co-transfected with the indicated Vif protein with either wild-type (LxxIxE) or alanine mutant (AxxAxA) B56 inhibitor peptide constructs. All experiments were repeated at least three independent times.

Previous studies have indicated that HIV-1 Vif uses a network of electrostatic interactions to recognize a conserved surface on B56α-ε proteins [13,14,20]. Given that MVV Vif exhibits a similar propensity to target all five B56 proteins for degradation, we were curious if it also recognized the same surface as HIV-1 Vif. To test this, we investigated an extensive panel of previously published and novel B56α single amino acid substitution mutants for resistance to HIV-1 and/or MVV Vif degradation activity (Figure 3). For generating novel variants, we started with previously identified interface residues and radiated outward targeting surface-exposed residues using the crystal structure of B56γ as a guide [13,20,24]. This resulted in the identification of a dozen amino acid substitution mutants that were resistant to HIV-1 and/or MVV Vif-induced degradation (Flow cytometry results depicted in Figure 3B, representative fluorescence microscopy images depicted in Figure 3C). While all 12 variants exhibited resistance to HIV-1 Vif-induced degradation, only five of these substitutions were resistant to MVV Vif. Amino acid substitution mutants G331R, E334R, E335K, E369K, and Y373W exhibited resistance to MVV Vif, all of which clustered to one surface and were mostly electronegative in composition (Figure 3A,B). Interestingly, four of the five residues are conserved among human and sheep B56α-ε proteins, which explains how MVV Vif can efficiently target human B56 proteins for degradation (G331 is only conserved in 3 of 5 human and sheep B56 proteins). Furthermore, degradation resistant variants separate into two distinct clusters, a larger surface resistant to HIV-1 Vif degradation and a smaller surface resistant to both HIV-1 and MVV Vif proteins (Figure 3A,B). 

To independently test the surface mapping results, experiments were performed using a previously characterized high-affinity peptide inhibitor [10,20,25]. This peptide contains a highly conserved LxxIxE motif that directly binds the substrate recognition groove of B56 proteins. Importantly, the wild-type peptide, but not a mutant derivative encoding an AxxAxA motif, has been shown to inhibit HIV-1 Vif-induced B56α degradation [20]. Based on our initial mapping experiments, we predicted that the wild-type peptide inhibitor would have a minimal impact on MVV Vif-induced degradation, as the B56 surface recognized is adjacent to the peptide binding groove. Consistent with previous observations, co-expression of a plasmid expressing four tandem copies of the wild-type peptide, but not the alanine peptide, could robustly inhibit HIV-1 Vif-induced degradation of B56α in a dose-dependent manner (Figure 3D). Importantly, co-expression of either the wild-type or alanine peptide with MVV Vif had a minimal impact on B56α degradation efficiency (Figure 3D). These separation-of-function results further support the model that HIV-1 and MVV Vif bind partially distinct B56 surfaces (Figure 3).

**Figure 4:**
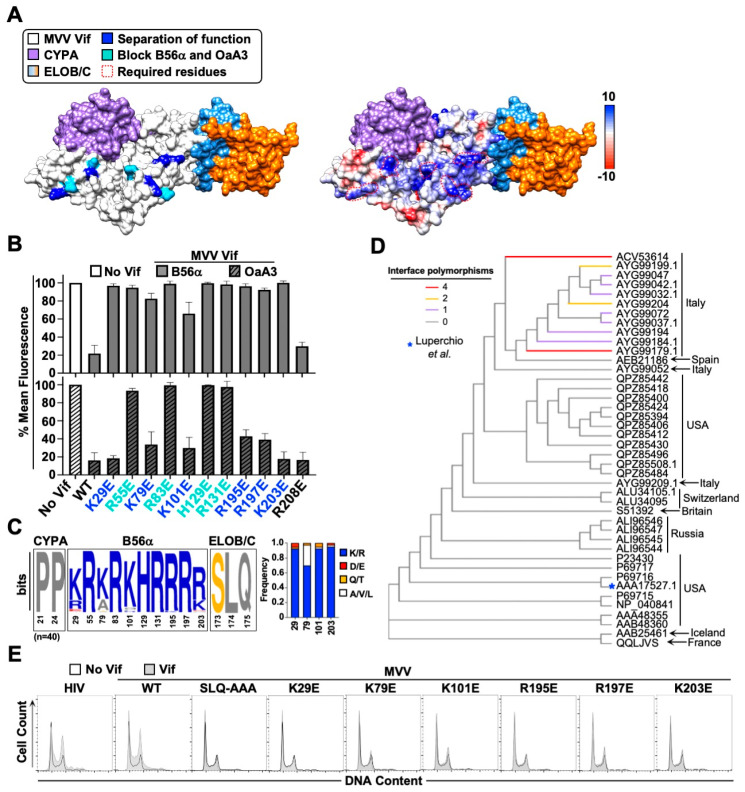
B56 antagonism and G/2M cell cycle arrest are conserved activities in global MVV Vif isolates. (**A**) Left, computational model of MVV Vif in complex with *CPYA* (PDB:1CWA) and ELOB/C (PDB:4N9F). Surface arginine or lysine residues that are required for MVV Vif-induced degradation of eGFP-B56α are colored based on separation-of-function (blue) or complete loss of degradation activity (cyan). Right, electrostatic surface potential map with red indicating negative charge, white indicating neutral charge, and blue indicating positive charge. Arginine and lysine residues are highlighted by dashed red outlines. (**B**) Quantification of eGFP mean fluorescence intensity from eGFP-B56α cells expressing the indicated MVV Vif proteins. (**C**) Weblogo of MVV Vif sequences downloaded from the NCBI database with amino acid residues required for the indicated protein–protein interactions highlighted (n = 40). Frequency of amino acid polymorphisms at all positions that exhibited variability are depicted as a bar graph (right). (**D**) Phylogenetic analysis of MVV Vif sequences with corresponding accession number and geographic region of isolation depicted. Coloring indicates the number of amino acid polymorphisms at positions required for MVV Vif-induced degradation of B56α-eGFP. Red, 4 amino acid polymorphisms; orange, 2 amino acid polymorphisms; purple, 1 amino acid polymorphism; grey, no polymorphisms. The blue asterisk highlights the MVV Vif isolate used in this study. (**E**) Flow cytometry histograms of representative cell cycle profiles of HeLa cells transiently expressing the indicated wild-type or mutant Vif proteins. All experiments were repeated at least three independent times.

Next, we wanted to determine the surface of MVV Vif used to recognize B56 substrates. Because the structure of MVV Vif has yet to be elucidated, we used computational modeling to generate a putative structure (Figure 4A, left). Before initiating structure guided mutagenesis experiments, we generated a partial cullin-RING degradation complex by superimposing *CPYA* (PDB: 1CWA) and elonginB/C (ELOB/C; PDB:4N9F) over their respective protein–protein interaction surfaces. Since the B56α surface recognized by MVV Vif was mostly electronegative, we reasoned that the corresponding Vif surface would be electropositive (Figure 4A, right). Using the *CPYA*-Vif-ELOB/C model as a guide, we generated charge swap substitution mutations at surface exposed arginine and lysine residues not sterically blocked by *CPYA* or ELOB/C binding and assessed degradation activity. This approach yielded a panel of MVV Vif separation-of-function mutants that were B56 degradation-deficient and A3 degradation-proficient, as well as several mutants that lost degradation activity against both substrates (Figure 4A,B). These results further confirm that the MVV Vif-B56 interaction surface is mediated through a network of electrostatic interactions.

We next wanted to estimate the proportion of MVV Vif isolates with the potential to degrade B56 proteins and induce G2/M cell cycle arrest in global circulation. All publicly available MVV Vif sequences were downloaded from the National Center for Biotechnology Information (NCBI) along with the country of sampling, and analyzed (n = 40). As expected, our analyses showed that residues required for binding *CPYA* (P21 and P24) and ELOB/C (173SLQ175) are completely conserved (Figure 4C). These analyses also indicated that MVV Vif amino acids required for B56 degradation and G2/M arrest occur at high frequencies globally, including the isolate from Greenwood et. al (Figure 4C,D) [7]. Interestingly, MVV Vif sequences that contained one or more amino acid polymorphisms within the putative B56 binding interface clustered phylogenetically and were isolated to the same geographic region (Figure 4D).

### 3.4. G2/M Cell Cycle Arrest and Conservation of the MVV Vif-B56 Interface 

Recent studies have established a direct cause-and-effect mechanism between HIV-1 Vif-induced degradation of B56 proteins and G2/M cell cycle arrest [13,14,17]. To determine if this causal relationship holds true for MVV Vif, we assessed the ability for wild-type and B56 degradation-deficient proteins to induce G2/M cell cycle arrest. For these experiments we focused on degradation defective mutants that exhibited separation-of-function activity to ensure MVV Vif proteins were stable and properly folded. As expected, both wild-type HIV-1 and MVV Vif proteins induced robust G2/M cell cycle arrest (Figure 4E). Importantly, all MVV Vif separation-of-function mutants failed to induce cell cycle arrest, further confirming the direct cause-and-effect relationship between B56 degradation and the subsequent G2/M cell cycle arrest phenotype (Figure 4E). Taken together, the conservation of these activities suggests that they are beneficial for lentiviral pathogenesis.

## 4. Discussion

While Vif’s canonical role in counteraction of A3 restriction factors has been extensively studied, it’s function in host phosphoproteome remodeling remains relatively unexplored. Here, we investigated the conservation of B56 antagonism and G2/M cell cycle arrest activities in diverse lentiviral Vif proteins. Vif isolates from non-primate lentiviral species were examined for B56 antagonism and only MVV Vif exhibited robust degradation activity. Further investigation revealed that MVV Vif could antagonize the entire family of B56α-ε proteins. Using structure-guided mutagenesis, the MVV Vif-B56 interface was mapped to distinct regions that form a network of electrostatic interactions. MVV Vif separation-of-function mutants were used to demonstrate that B56 degradation and G2/M cell cycle arrest are inextricably linked, which supports recent conclusions examining B56 antagonism and G2/M cell cycle arrest induced by HIV-1 Vif [13,14,17]. Phylogenetic analyses indicate that these activities are likely highly conserved in global MVV Vif isolates, which is consistent with previous observations regarding the conservation of this activity in HIV-1 and SIV isolates [7,13,14,20]. Taken together, these observations suggest that B56 antagonism and G2/M cell cycle arrest are likely advantageous for lentiviral pathogenesis. 

The conservation of B56 degradation and G2/M cell cycle arrest activity in MVV Vif is somewhat surprising given the limited sequence homology to HIV-1 Vif (only about 15% identity, with the longest stretch of homology being the six residue ELOB/C binding motif) (Figure 1 and Figure 4). Comparisons between the HIV-1 Vif crystal structure and the MVV Vif computational model also demonstrate a lack of conservation, with little-to-no structural homology between proteins. While HIV-1 and MVV Vif sequences and structures share little homology, the biophysical properties of the HIV-1 and MVV Vif-B56 interfaces exhibit remarkable similarities. Extensive mutagenesis experiments revealed that both the HIV-1 and MVV Vif proteins recognize clusters of conserved negatively charged amino acid residues on the surface of B56 proteins (Figure 3). While HIV-1 Vif sterically blocks the B56 substrate binding groove and MVV Vif does not, binding of either viral protein to B56 proteins would interfere with cellular substrate recognition prior to degradation. Recent studies have demonstrated that B56 substrate binding requires the LxxIxE binding pocket as well as electrostatic interactions that occur adjacent to the peptide binding cleft, encompassing residues E335 and D338 [26]. These residues are of interest since G331, E334, and E335 are required for MVV Vif-induced degradation, indicating that both HIV-1 and MVV Vif proteins use a dual mechanism to inhibit PP2A activity (i.e., degradation and inhibition of B56-substrate binding) (Figure 3).

As is the case for HIV-1 Vif, MVV Vif utilizes an extensive surface of electropositive residues to specifically recognize B56 substrates (as demonstrated by separation-of-function mutants K29, K79, K101, R195, R197, and K203) (Figure 4). Importantly, MVV Vif separation-of-function mutants deficient for B56 degradation activity also fail to induce G2/M cell cycle arrest, further supporting the direct cause-and-effect model postulated previously. Finally, it is worth noting that while the other non-primate lentiviral Vif isolates examined in this study did not induce B56α degradation, previous studies estimate this activity is present in roughly 30–50% of HIV-1 isolates in global circulation [13,14,20]. Therefore, it is possible that further exploration of Vif isolates from each species could yield variants that exhibit B56 antagonism.

Phylogenetic analysis of MVV Vif isolates indicate that B56 antagonism and G2/M cell cycle arrest activity are likely highly conserved globally (Figure 4). While the number of MVV Vif sequences available for analysis is limited, our findings are in line with previous analyses using a more extensive database of HIV-1 Vif isolates [13,14]. While the importance of PP2A antagonism has yet to be established in vivo, several observations have been made that suggest it could be beneficial for HIV-1 pathogenesis. For example, PP2A-B56 complexes regulate protein translation kinetics, secretion of pro-inflammatory cytokines, and T cell activation, all of which could impact nascent particle production, virus replication kinetics, or suppression of host immune responses [27,28,29,30]. Furthermore, subversion of the host cell cycle and antagonism of PP2A-B56 complexes are conserved activities among diverse viral pathogens, including human T-lymphotropic virus, Ebola virus, adenovirus, infectious bronchitis virus, human polyoma virus, and simian virus 40 [31,32,33,34,35,36,37]. Taken together, these observations suggest that remodeling of the cellular phosphoproteome and induction of G2/M cell cycle arrest are likely advantageous for lentiviral pathogenesis.

## 5. Conclusions

B56 degradation and G2/M cell cycle arrest activities are ancient and conserved functions of lentiviral Vif proteins. Whether these functions arose from a common ancestor, or emerged independently, conservation of these activities strongly suggests that they are advantageous for lentiviral pathogenesis. 

## Data Availability

Not applicable.
